# Effect of Mixing Ratio of Oppositely Charged Block Copolymers on Polyion Complex Micelles for In Vivo Application

**DOI:** 10.3390/polym13010005

**Published:** 2020-12-22

**Authors:** Noriko Nakamura, Yuki Mochida, Kazuko Toh, Shigeto Fukushima, Horacio Cabral, Yasutaka Anraku

**Affiliations:** 1 Department of Bioengineering, Graduate School of Engineering, The University of Tokyo, 7-3-1 Hongo, Bunkyo-ku, Tokyo 113-8656, Japan; nnakamura@bmw.t.u-tokyo.ac.jp; 2 Innovation Center of NanoMedicine, Kawasaki Institute of Industrial Promotion, 3-25-14 Tonomachi, Kawasaki-ku, Kawasaki 210-0821, Japan; mochida-y@kawasaki-net.ne.jp (Y.M.); tou-k@kawasaki-net.ne.jp (K.T.); fukushima-s@kawasaki-net.ne.jp (S.F.)

**Keywords:** polyion complex micelles, mixing charge ratio, core formation, blood circulation, biodistribution

## Abstract

Self-assembled supramolecular structures based on polyion complex (PIC) formation between oppositely charged polymers are attracting much attention for developing drug delivery systems able to endure harsh in vivo environments. As controlling polymer complexation provides an opportunity for engineering the assemblies, an improved understanding of the PIC formation will allow constructing assemblies with enhanced structural and functional capabilities. Here, we focused on the influence of the mixing charge ratio between block aniomers and catiomers on the physicochemical characteristics and in vivo biological performance of the resulting PIC micelles (PIC/m). Our results showed that by changing the mixing charge ratio, the structural state of the core was altered despite the sizes of PIC/m remaining almost the same. These structural variations greatly affected the stability of the PIC/m in the bloodstream after intravenous injection and determined their biodistribution.

## 1. Introduction

With the progress of polymer technology, self-assembled supramolecular structures of precisely designed polymers have been receiving much attention for fundamental studies [1,2] and the development of functional biomaterials [3,4]. Among supramolecular assemblies, polyion complexes (PICs), which are formed by electrostatic interaction between oppositely charged polymers in aqueous conditions without using organic solvents, have shown exceptional features for constructing biomaterials with controlled behavior at the biointerface and wide-range applicability, such as drug delivery systems (DDS) [5,6,7], bioimaging [8] and artificial organelles [9].

PIC structures are versatile biomaterials with the ability to obtain different physical properties based on the same polymer combination just by changing the surroundings, i.e., the ionic strength of solvents [10,11] and environmental pH [12], as well as by controlling parameters of the polymers, including the degree of polymerization of the charged segments [13], the polymer concentration and the mixing charge ratio [14]. For example, tuning the chain length of the charged segments and the ionic strength of solvents resulted in PIC structures with different states in a solution, precipitates or forming coacervates [15,16]. Nevertheless, effective control of PIC assembly is challenging, and slight variations in the abovementioned factors may lead to catastrophic changes in the nanostructures.

Kataoka et al. have lay the foundations for constructing a wide variety of PIC structures with nano- and micron-sized architectures from block aniomers and block catiomers consisting of poly(ethylene glycol) (PEG) as a hydrophilic segment and polyamino acids as the charged blocks [7,14,17,18], which have been effectively used as nanocarriers for drug and gene delivery. The structure of these assemblies can be controlled by engineering the dimensional influence of the charged segment [19], the weight fraction of PEG [20] and the ionic strength [6]. Herein, we extended the understanding of PIC formation by focusing on the effect of the mixing charge ratio of PEG-catiomers and PEG-aniomers having relatively short PEG strands of 2 kDa, which is shorter than the PEG block of most polymeric micelles developed in our group [21], on the assembly of core–shell PIC micelles (PIC/m). Such relatively short PEG segments may lead to alterations in PIC/m due to their lower steric hindrance compared to longer PEG blocks. The structural changes of the micelles and the accompanying in vivo behavior were assessed to determine the influence of the mixing charge ratio.

## 2. Results and Discussion

**Scheme 1:**
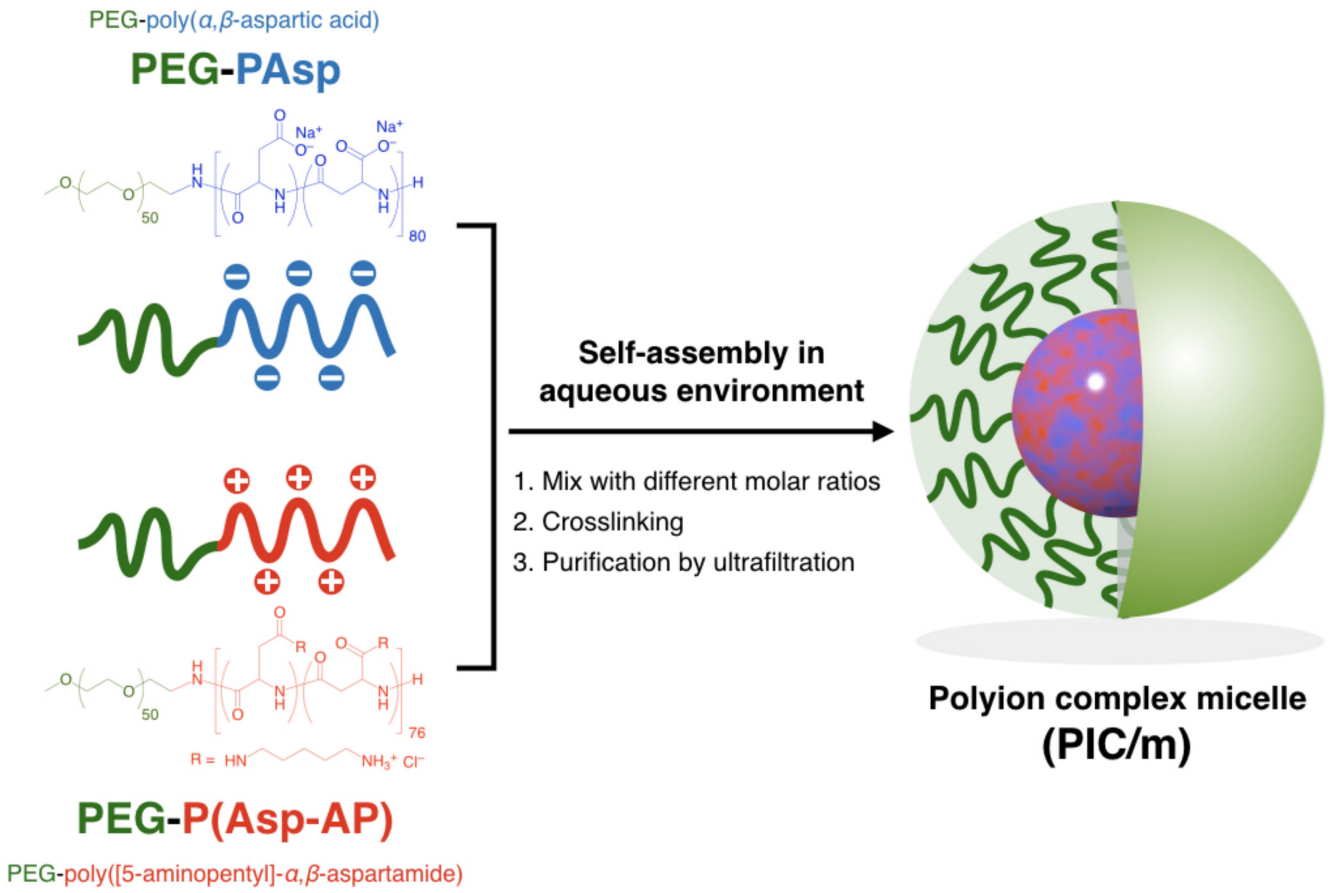
Scheme of polyion complex micelles (PIC/m) preparation via the assembly of oppositely charged block copolymers with different mixing molar ratios of C/A.

**Figure 1:**
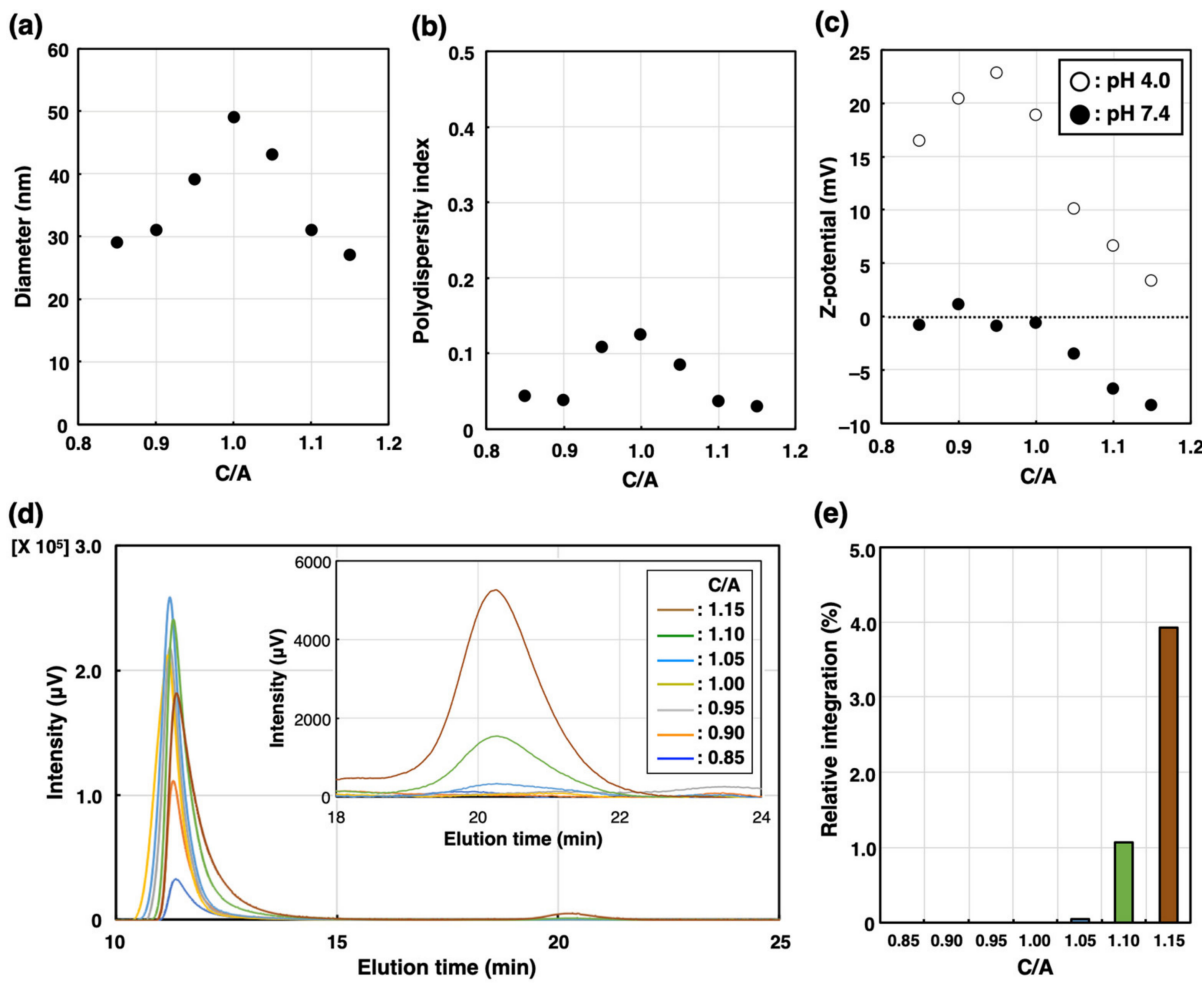
Characterization of PIC/m prepared with different mixing molar ratios of C/A in the range from 0.85 to 1.15 in 10 mM phosphate buffer (0 mM NaCl). (**a**) Diameter, (**b**) polydispersity index and (**c**) Z-potential (open circles denote pH = 4.0, closed circles denote pH = 7.4) are determined by DLS. (**d**) Chromatogram traces of PIC/m were measured by SEC. Brown, green, light blue, yellow, gray, orange and blue colored lines indicate C/A = 0.85, 0.90, 1.00, 1.05, 1.10 and 1.15, respectively. (**e**) Relative peak area of block copolymers (elution peak around 21 min) normalized to micelle peak area to that of PIC/m (elution peak around 11 min).

The building units of PIC/m, i.e., PEG-poly(*α*,*β*-aspartic acid) (PEG-PAsp; *M*_n_ of PEG = 2200, DP of P (Asp) = 80), PEG-poly([5-aminopentyl]-*α*,*β*-aspartamide) (PEG-P(Asp-AP); *M*_n_ of PEG = 2200, DP of P(Asp-AP) = 76), were synthesized according to our previous method (for the characterization of these block copolymers, see the Supplementary Information) [19]. PIC/m were prepared by combining solutions containing 1 mg/mL of the oppositely charged block copolymers mentioned above in a 10 mM phosphate buffer (pH 7.4) (Scheme 1). The features of the micelles were identified by a dynamic light scattering (DLS) measurement. Samples with mixing molar ratios of [carboxylate]/[amine] ([mol]/[mol], C/A) in the range from 0.85 to 1.15 resulted in narrow distributed micelles with diameters ranging from 30 nm to 50 nm (Figure 1a,b). The carboxylate and amine units of the charged segments were cross-linked by 1-ethyl-3-(3-dimethylamino-propyl) carbodiimide (EDC), followed by ultrafiltration to remove the excess EDC and free polymers. The surface Z-potential of each micelle was determined by an electrophoretic light scattering (ELS) measurement. It has been reported that the surface potential of nanoparticles is attenuated after PEGylation due to the steric effects of PEG chains [22,23]. In our case, the PIC/m with an excess number of anionic-charged block copolymers showed decreasing Z-potential depending on the number of additional carboxylate units regardless of PEGylation. On the other hand, the Z-potential of PIC/m with excess cationic-charged block copolymers was not affected much (Figure 1c). This result seems to be independent of the ionization ratio of carboxylates and amines in the buffer, as the trend of Z-potential was similar in a 10 mM acetate buffer (pH = 4.0). Thus, we hypothesized that the excess of carboxylate or amine units may have an effect on the structure of PIC/m.

The number of block copolymers that did not participate in micelle formation was quantified using size exclusion chromatography (SEC) before purification. As shown in Figure 1d, PIC/m were detected at 11 min regardless of C/A in a range from 0.85 to 1.15, whereas the block copolymers that were not integrated into the micelles were only detected for C/A = 1.10 and C/A = 1.15 at an elution time of 21 min. Additionally, the ratio of the area of the peak of block copolymers to that of PIC/m was found to be larger for C/A = 1.15 than for C/A = 1.10. Thus, whilethe extra anionic-charged block copolymers were excluded from micelle formation, excess cationic-charged block copolymers could participate in the formation of PIC/m against unfavorable charge mismatching. On the other hand, previous observations by Kataoka et al. have shown that PIC/m assembled from PEG-PAsp aniomers and PEG-P(L-lysine) block catiomers having relatively longer PEG strands with a molecular weight of 5 kDa, and anionic and cationic segments with matching lengths achieve charge neutralization in the core. This contradictory behavior might be explained by the shorter PEG length used in our study, i.e., 2 kDa, which would result in less steric repulsion and phase mixing between the core-forming blocks and the PEG segments, leading to the incorporation of extra catiomers into the PIC/m structure.

To further understand the formation of the PIC/m, we evaluated how C/A affected other structural characteristics. Thus, we first looked into the association number of each PIC/m assembled in 10 mM phosphate buffer (0 mM NaCl), which was quantified by identifying the molecular weight through static light scattering (SLS). The association number of PIC/m with a stoichiometric charge balance (C/A = 1.0) was found to be 400 polymers, which is four-fold higher than that of PIC/m at C/A = 0.90, i.e., 97 polymers, and about twice higher than that of PIC/m at C/A = 1.10, i.e., 177 polymers (Table 1). Moreover, from the results of the static light scattering measurement, it is suggested that the micellar association number decreased when the PIC micelles formed at charge mismatching followed by a decrease in the size of the core. These results suggest that the PIC/m may be forming different core–shell structures depending on C/A. 

**Figure 2:**
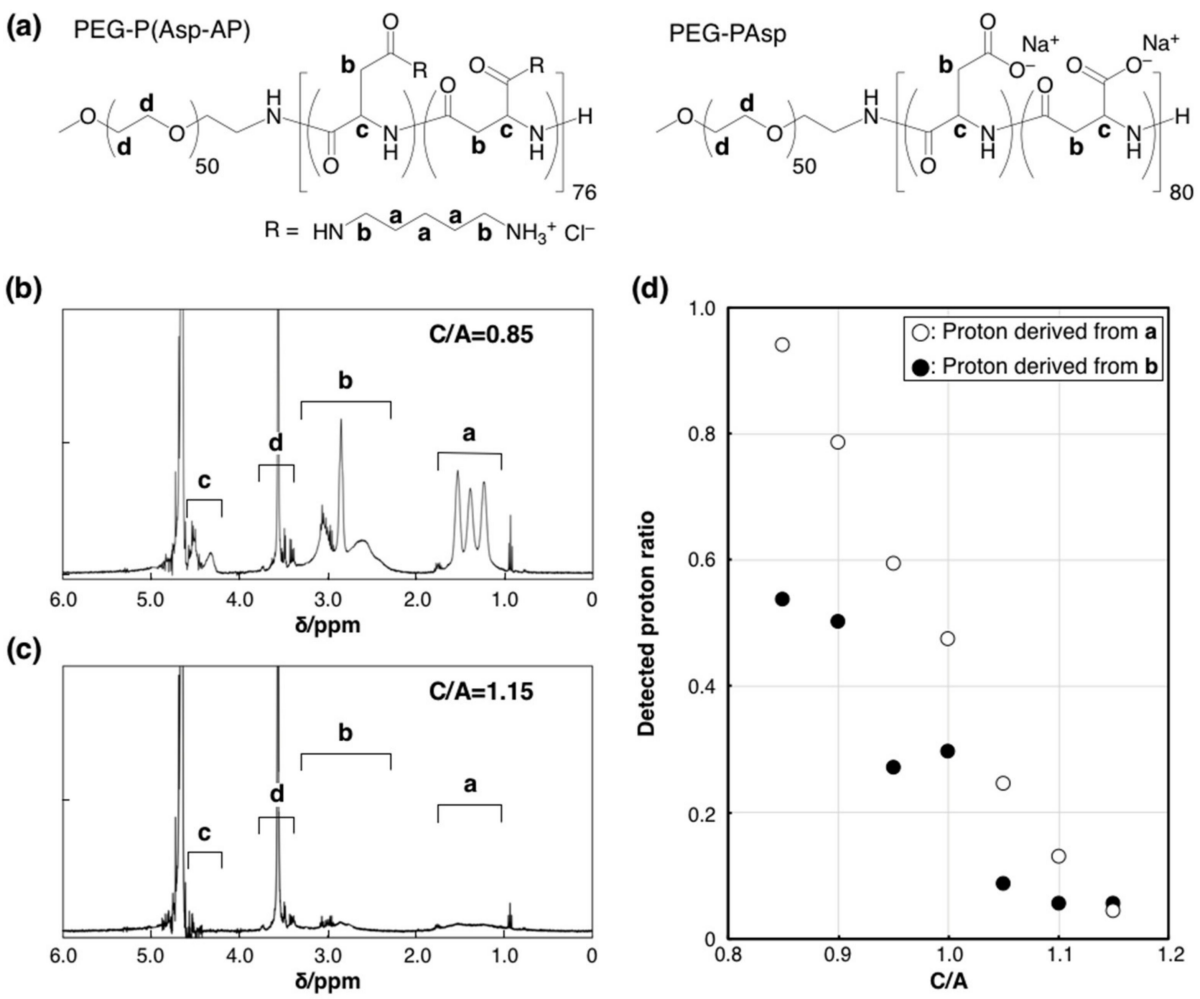
^1^H-NMR analysis of the PIC/m core. (**a**) Chemical structures of PEG-P(Asp-AP) and PEG-PAsp. ^1^H-NMR spectra of PIC/m prepared with a mixing ratio of C/A (**b**) at 0.85 and (**c**) 1.15 (10 mM deuterium phosphate buffer (pD = 7.0), 400 MHz, 37 °C). a, b, c and d denote each proton of chemical structures in (**a**). (**d**) Detected proton ratio between PIC/m and polymers (open and closed circles denote proton derived from “a” and “b” of (**a**).

Further structural analysis of the charged PIC/m core was conducted by proton nuclear magnetic resonance (^1^H-NMR). PIC/m are considered to be composed of a core (represented by the assembled charged part of the polymer) and a hydrophilic shell (formed by the hydrated PEG segment of the block copolymer). Thus, the formation of micelles is expected to weaken the ^1^H-NMR signals from protons located within the micellar core due to restricted internal molecular motion. In fact, it has been reported that the peaks from protons of molecules encapsulated in the core of micelles showed significantly decreased intensity [24,25,26]. The ^1^H-NMR measurements were made with cross-linked PIC/m prepared in a 10 mM deuterium phosphate buffer (pD = 7.0). The intensities of the peaks attributed to the charged segments were normalized to that of PEG (Figure 2a–c). These normalized intensities decreased when C/A increased from 0.85 to 1.15 (Figure 2d), indicating higher intermolecular mobility in the core of PIC/m with excess cationic-charged block copolymer. Moreover, the cross-linking of the PIC/m was estimated by analyzing the remaining -COO^−^ groups in the micelles by Fourier-transform infrared spectroscopy (FT-IR) measurement, as previously reported [27]. The FT-IR results (Figure S5) indicated that the peak of remaining -COO^−^ groups in the PIC/m increased as C/A decreased, which implies that less -COO^−^ groups have been consumed during the cross-linking reaction despite the increasing number of amines in catiomer-rich PIC/m. This behavior might be related to the inadequate segregation of the charged blocks at C/A higher than 1 when using PEG strands of 2 kDa. 

**Figure 3:**
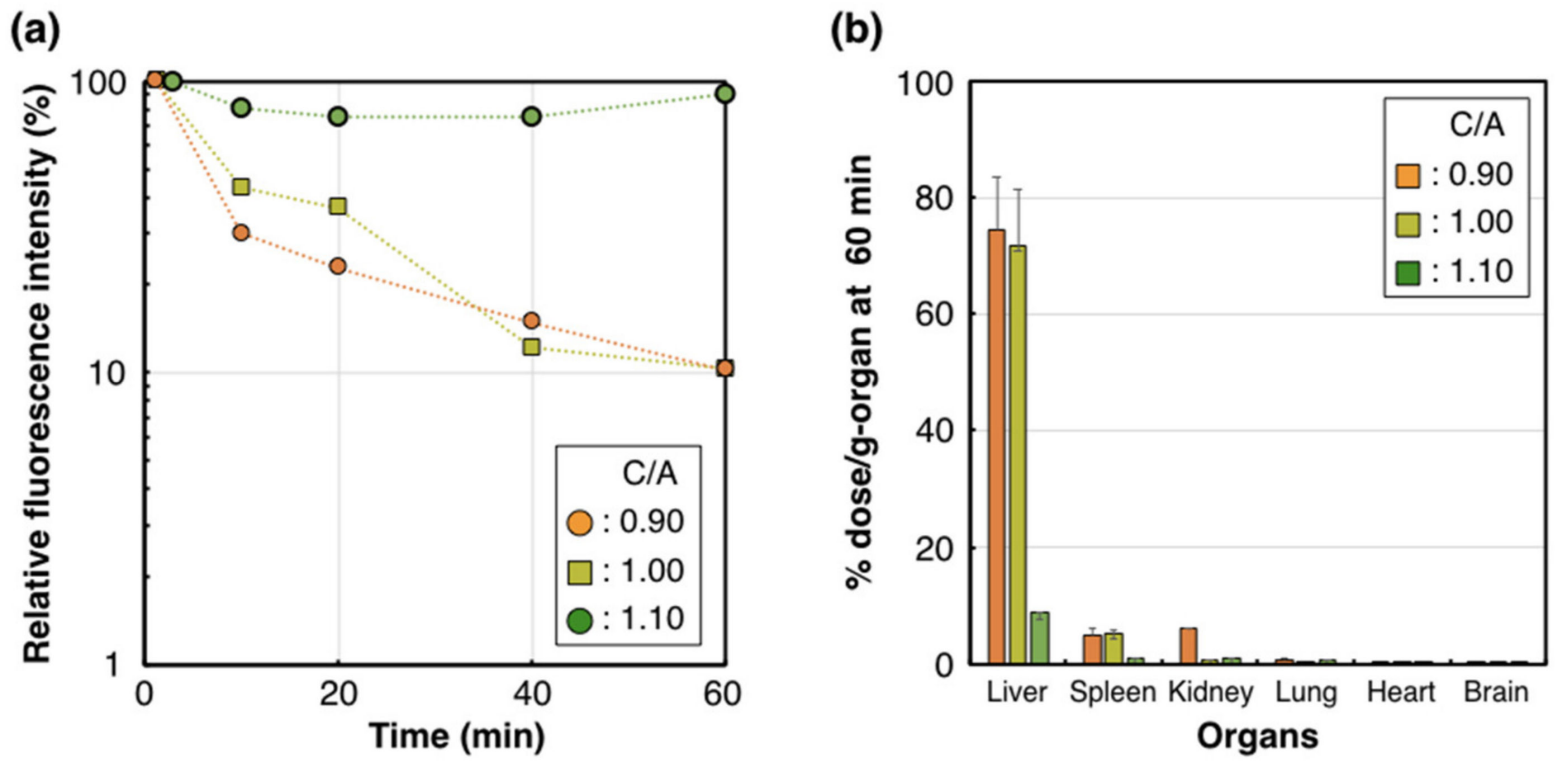
Pharmacokinetic profile of the Cy5-PIC/m with a C/A of 0.90, 1.00 and 1.10. (**a**) Plasma retention of Cy5-PIC/m (orange circle, yellow square and green circle indicate 0.90, 1.00 and 1.10, respectively) observed by IVRT-CLSM. (**b**) Accumulation of Cy5-PIC/m in the liver at 60 min. *n* = 4. Data are shown as means ± SEM.

To determine the influence of these structural variations on the biological performance of PIC/m, we evaluated the stability of PIC/m in the bloodstream, as well as their organ distribution in mice, which are significant features for applying PIC/m as nanocarriers. Cy5-labeled PIC/m (Cy5-PIC/m) were prepared by conjugating fluorescent molecules to the ω-end of the cationic-charged block copolymers. The Cy5-PIC/m prepared with a C/A of 0.90, 1.00 and 1.10 were evaluated in vivo, as follows: The Cy5-PIC/m were intravenously (i.v.) injected into mice, and the fluorescent intensity in the vein was continuously monitored by intravital real-time confocal laser scanning microscopy (IVRT-CLSM) [28]. The results showed that PIC/m with a C/A of 0.90 and 1.00 disappeared from the bloodstream almost immediately after the administration, and only 10% of the injected dose was detectable 60 min after administration (Figure 3a). On the other hand, 90% of injected PIC/m with a C/A of 1.10 circulated in the bloodstream stably (Figure 3a). These results confirmed that the excess amounts of amines in the core of PIC/m decreases the blood circulation of the micelles. The organ distribution (liver, spleen, kidney, lung, heart and brain) of the PIC/m was evaluated at 60 min after intravenous injection by quantifying the fluorescent intensity of each organ after homogenization (Figure 3b). PIC/m remaining in the bloodstream were removed by perfusing with D-PBS (–) from the hepatic portal vein before collecting organs. While Cy5-PIC/m with a C/A of 0.90 and 1.00 showed high accumulation in the liver, spleen and kidney (Figure 3b), Cy5-PIC/m with a C/A of 1.10 presented reduced distribution to the organs, showing levels lower than 1% dose/g-organ. This result is consistent with the longevity of Cy5-PIC/m with a C/A of 1.10 in the bloodstream (Figure 3a).

**Figure 4:**
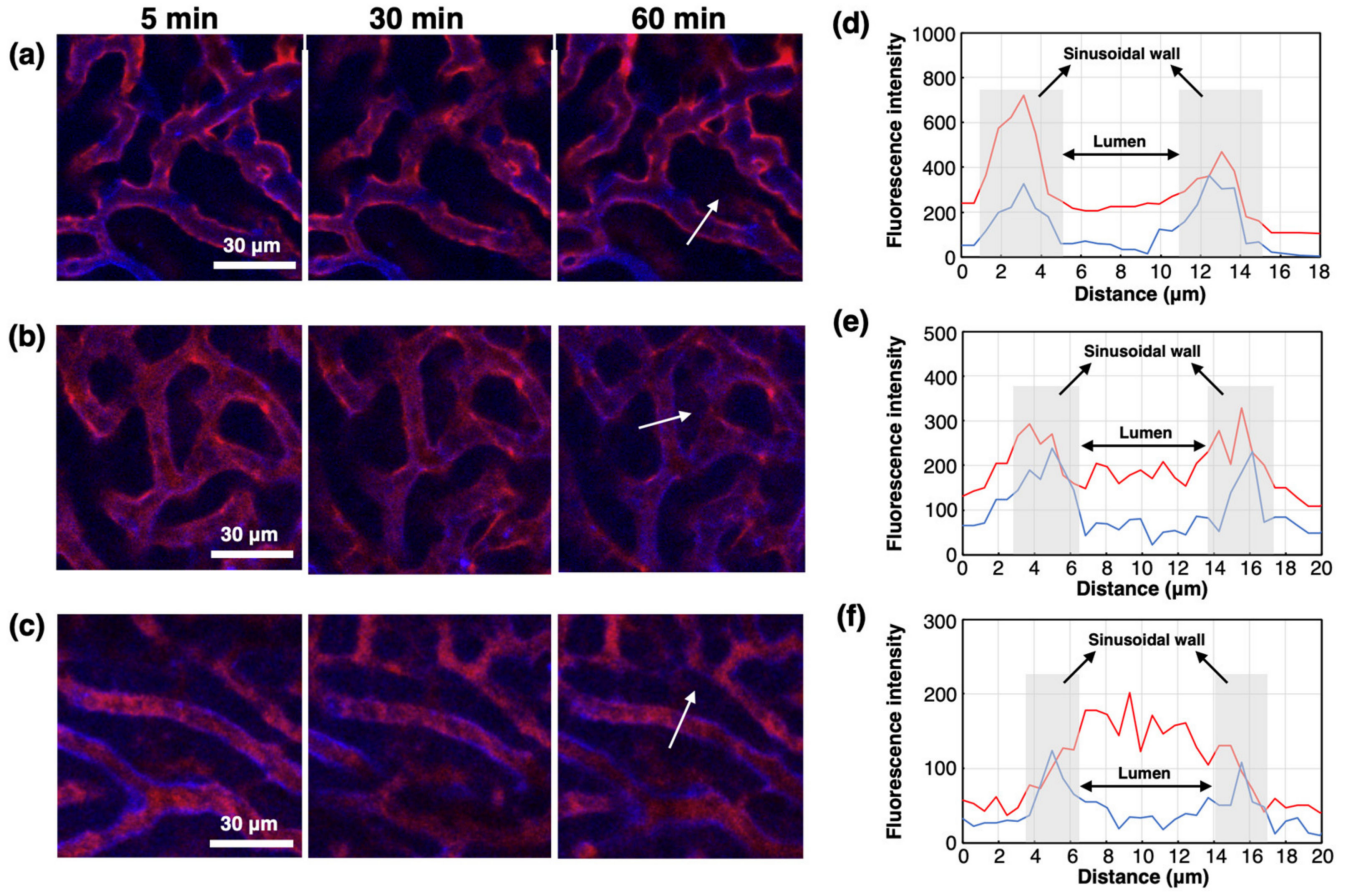
Attachment of Cy5-PIC/m with a C/A of 0.90, 1.00 and 1.10 to the liver sinusoidal wall. IVRT-CLSM images after injection of Cy5-PIC/m (red) with a C/A of (**a**) 0.90, (**b**) 1.00 and (**c**) 1.10. Blue color indicates the liver sinusoidal wall (endothelial cell) stained by eFluor450-PECAM-1. Intensity profiles of Cy5 (red line) and eFluor450 (blue line) in the white arrows in (**a**), (**b**) and (**c**) are shown in (**d**), (**e**) and (**f**), respectively: (d) 0.90; (e) 1.00; (f) 1.10.

As PIC/m with a C/A of 0.90 and 1.00 showed the highest accumulation in the liver, the microdistribution of Cy5-PIC/m in the liver was examined in real-time by intravital microscopy. For direct observation, the liver was surgically exposed and glued directly to the cover glass using a drop of oil [29]. Endothelial cells in the liver sinusoidal wall were stained by i.v. injection of eFluor450-PECAM-1 at 30 min before starting IVRT-CLSM observation. PIC/m with a C/A of 1.10 showed a high fluorescent signal derived from PIC/m in the vascular lumen during the observation period (Figure 4c,f), whereas PIC/m with a C/A of 0.90 and 1.00 were observed binding along the vessel wall and rapidly clearing from the bloodstream, with PIC/m having a C/A of 0.90 being cleared most rapidly (Figure 4a,b,d,e). From our ^1^H-NMR and SEC observations, it is reasonable to assume that the PIC/m with a C/A of 0.90 and 1.00 may be exposing extra cationic units in the PIC core, Moreover, at pH 7.4, there are more protonated amines in the block catiomers than deprotonated carboxylates in the block aniomers [30]. Thus, the binding of PIC/m with a C/A of 0.90 and 1.00 to the liver sinusoidal wall could be attributed to the abundant anionic proteoglycans present on the sinusoidal extracellular matrix, which can capture oligocations [29], as well as the high expression of scavenger receptors, which recognize cationic macromolecules, on sinusoidal cells [31].

## 3. Conclusions

Monodispersed PIC/m with diameters around 30–50 nm were effectively assembled in a wide C/A ratio across stoichiometric conditions by using catiomers and aniomers having PEG strands of 2 kDa. Although the sizes of the resulting PIC/m are comparable, they present different physical properties, such as Z-potential and micellar association number that significantly altered the biological performance. Thus, PIC/m prepared with high C/A showed long blood circulation, whereas PIC/m with low C/A were rapidly cleared from the bloodstream and accumulated mainly in the liver. Microscopic studies by direct liver observation with IVRT-CLSM indicated that PIC/m with low C/A binds to the liver sinusoidal wall, which express abundant anionic proteoglycans. These findings provide valuable knowledge for the precise formation of PIC-based nanocarriers with desired performance in vivo.

## Figures and Tables

**Table 1:** Structural characteristics features of PIC/m prepared with different mixing molar ratios of C/A.

C/A	0.90	0.95	1.00	1.05	1.10
Diameter (nm) *	31	39	49	43	31
Micellar association number **	97	286	400	185	177

* Values determined by DLS measurements. ** Values determined by SLS measurements.

## Data Availability

The data presented in this study are available on request from the corresponding author.

## Supplementary Materials

The following are available online at https://www.mdpi.com/2073-4360/13/1/5/s1, Figure S1: ^1^H-NMR spectrum of PEG-PAsp, Figure S2: GPC chromatogram of PEG-PAsp, Figure S3: ^1^H-NMR spectrum of PEG-P(Asp-AP), Figure S4: GPC chromatogram of PEG-P(Asp-AP). Figure S5. FT-IR FT-IR spectra of PIC/m in 10 mM deuterium phosphate buffer.
