# Supplementary material for: Effect of Mixing Ratio of Oppositely Charged Block Copolymers on Polyion Complex Micelles for In Vivo Application
Source: Polymers (Basel). 2020 Dec 22;13(1):5. doi: 10.3390/polym13010005 (PMC7792805; doi:10.3390/polym13010005)
Supplement: Supplementary file 1 [file polymers-13-00005-s001.pdf]

Supplementary Information

Effect of mixing ratio of oppositely charged block copolymers on polyion complex micelles for *in vivo* application

Noriko Nakamura[1,2,a], Yuki Mochida [2,a], Kazuko Toh[2], Shigeto Fukushima[2], Yasutaka Anraku[1,2*] and Horacio Cabral[1,2*]

1 Department of Bioengineering, Graduate School of Engineering, The University of Tokyo, 7-3-1 Hongo, Bunkyo-ku, Tokyo 113-8656, Japan.
2 Innovation Center of NanoMedicine, Kawasaki Institute of Industrial Promotion, 3-25-14 Tonomachi, Kawasaki-ku, Kawasaki 210-0821, Japan.
* Correspondence should be addressed to Y. Anraku (e-mail: anraku@bmw.t.u-tokyo.ac.jp) or to H. Cabral (e-mail: horacio@bmw.t.u-tokyo.ac.jp)

1. Materials

Poly(ethylene glycol)-poly(*β*-benzyl-L-aspartate) (PEG-PBLA, M_n of PEG = 2,200, degree of polymerization (DP) of P(BLA) = 80) was synthesized as previously reported [1]. Dichloromethane (DCM) were purchased from Kanto Chemical Co. Inc. (Tokyo, Japan). Hexane, ethyl acetate and deuterium oxide were purchased from Sigma Aldrich Japan Co. LLC (Tokyo, Japan). *N*-methyl-2-pyrrolidone (NMP) and D-PBS(-) were purchased from FUJIFILM Wako Pure Chemical Co. (Tokyo, Japan). 1,5-diaminopentane (DAP) and 1-ethyl-3-(3-dimethylaminopropyl) carbodiimide hydrochloride (EDC/HCl) were purchased from Tokyo Chemical Industry Co. Ltd. (Tokyo, Japan). NMP and DAP were distilled over CaH_2 prior to use. Sulfo-cyanine 5 succinimidyl ester (sulfo-cy5-NHS ester) was purchased from Lumiprobe Co. (Hunt Valley, MD, USA). Dimethyl sulfoxide (DMSO) was purchased from Nacalai Tesque Inc. (Kyoto, Japan). Passive lysis buffer was purchased from Promega Co. (Madison, WI, USA). Isoflurane was purchased from Abbott Japan Co. Ltd., Tokyo, Japan). eFuor450-conjugated CD31 (PECAM-1) monoclonal antibody was purchased from Thermo Scientific (Waltham, MA, USA).

2. Synthesis and characterization of polymer

Synthesis of PEG-poly(α,β-aspartic acid)

PEG-poly(*α,β*-aspartic acid) (PEG-PAsp) was synthesized by deprotection of the benzyl ester groups of PEG-PBLA. PEG-PBLA was dissolved in 0.25 M NaOH (5 molar equivalent to benzyl group) and stirred at room temperature for 2 h to hydrolyze the benzyl ester moiety. The reaction mixture was dialyzed against deionized water using a Specra/Por 1 dialysis membrane (Repligen,

Waltham, MA, USA) [MWCO: 6,000 – 8,000 Da] for 2 days, and then lyophilized subsequently.

The DP of P(Asp) was calculated to be 80 from the ^{1}H-NMR measurement by comparing the peak area ratio using the methylene protons of PEG as the reference peak (**Figure S1.**). The GPC trace of the obtained PEG-PAsp was unimodal (**Figure S2.**).

Synthesis of PEG-poly([5-aminopenthyl]-α,β-aspartamide)

PEG-poly([5-aminopenthyl]-*α*,*β*-aspartamide) (PEG-P(Asp-AP)) was synthesized by the deprotection of the benzyl ester groups of PEG-PBLA by ester-amide exchange reaction. Firstly, lyophilized PEG-PBLA was dissolved in NMP (13.3 mg/mL). A mixture of NMP and DAP (100 molar equivalent to BLA moiety) was added to the solution, and then stirred at 12 ℃ for 1 h respectively. The reaction solution was neutralized with excess amount of 1 M HCl in ice bath. The resulting solution was dialyzed against 10 mM HCl for 1 day and subsequently against deionized water for 2 days using a Specra/Por 1 dialysis membrane [MWCO: 6,000 – 8,000 Da]. PEG-P(Asp-AP) was given as a chloride salt after the lyophilization.

The DP of P(Asp-AP) unit was calculated to be 76 from the ^{1}H-NMR measurement by comparing the peak area ratio using the methylene protons of PEG as the reference peak (**Figure S3.**). The GPC trace of the obtained PEG-P(Asp-AP) was nearly unimodal (**Figure S4.**).

Synthesis of Cy5-labeled PEG-P(Asp-AP)

The ω-terminal end of PEG-PBLA was labeled with sulfo-Cy5-NHS ester by stirring in DMSO (50 mg/mL) at room temperature overnight. The resulting solution was reprecipitated with mixture of hexane and ethyl acetate (3:2, v/v), followed by a filtration under reduced pressure. The obtained PEG-PBLA-Cy5 was reacted with DAP according to the former section, and then Cy5-labeled PEG-P(Asp-AP) was obtained.

Polymer characterization

The degree of polymerization (DP) was determined by proton nuclear magnetic resonance (^{1}H-NMR) spectroscopy using JEOL ECS400 (JEOL Ltd., Tokyo, Japan) at 400 MHz. The molecular weight distribution of block copolymers was verified gel permeation chromatography (GPC) (JASCO, Tokyo, Japan) equipped with a Superdex 200-10/300GL column (GE Healthcare, Chicago, IL, USA).

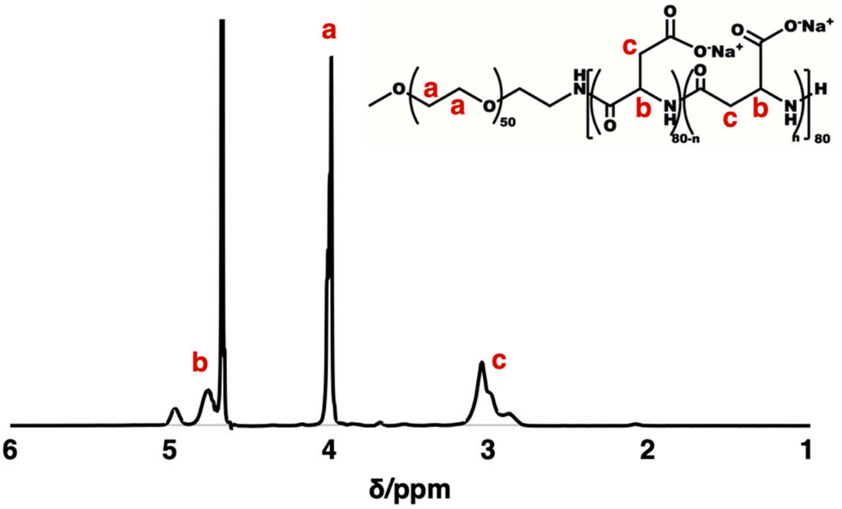

Figure S1. ^{1}H-NMR spectrum of PEG-PAsp (solvent: D_2O, temperature: 80 ℃)

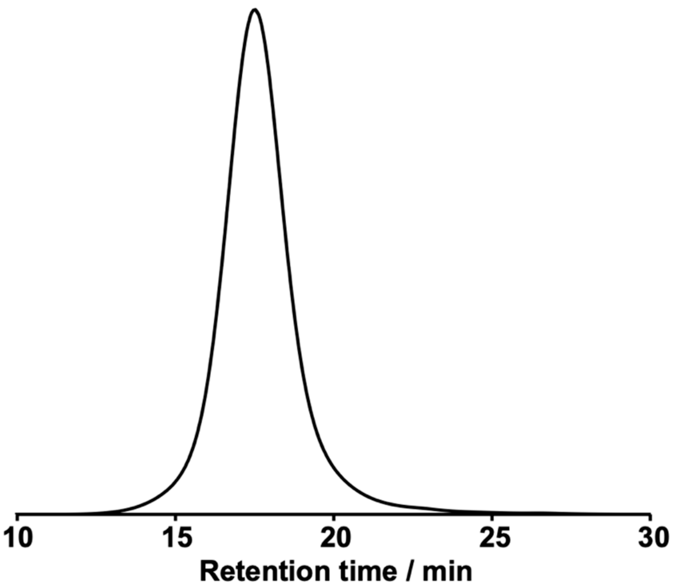

Figure S2. GPC chromatogram of PEG-PAsp (flow rate: 0.75 mL/min, eluent: 10 mM PB (pH 7.4), 150 mM NaCl, detector: UV at 220 nm)

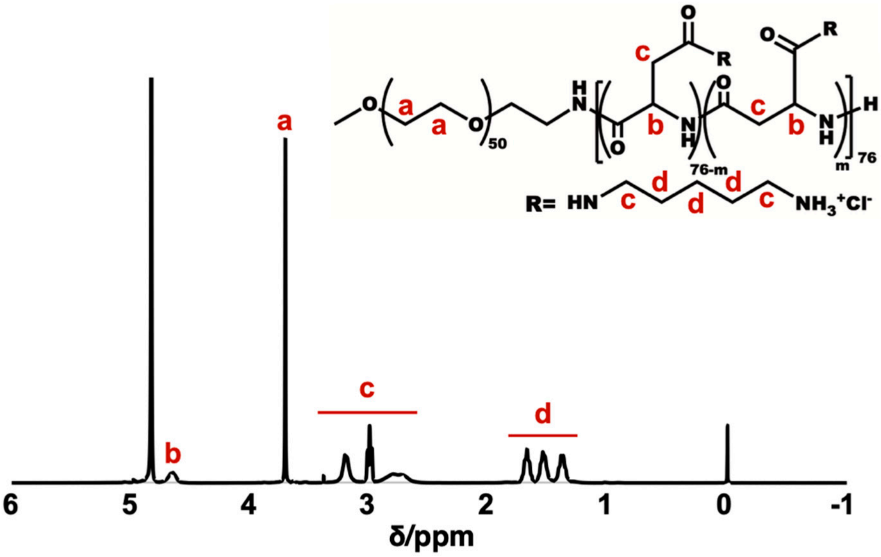

Figure S3. ^{1}H-NMR spectrum of PEG-P(Asp-AP) (solvent: D_2O, temperature: 25 ℃)

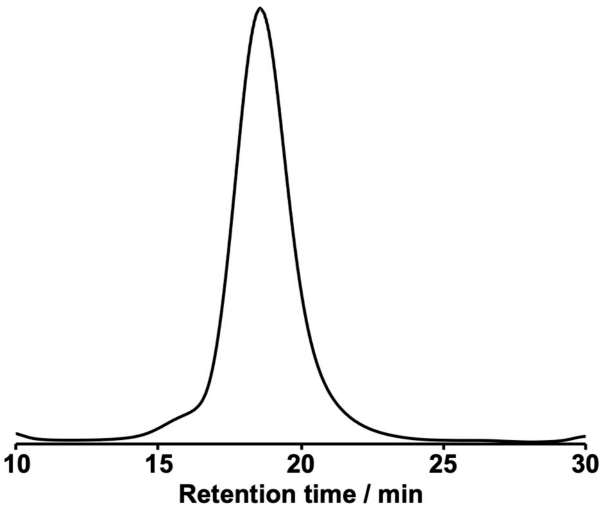

Figure S4. GPC chromatogram of PEG-P(Asp-AP) (flow rate: 0.75 mL/min, eluent: 10 mM Acetic acid, 500 mM NaCl, detector: UV at 220 nm)

3. Preparation and characterization of polyion complex micelle (PIC/m)

Preparation of PIC/m

PEG-PAsp and PEG-P(Asp-AP) (and PEG-P(Asp-AP)-cy5 for the preparation of cy5-labeled PIC/m) were dissolved in 10 mM phosphate buffer (PB, pH 7.4, 0 mM NaCl) separately to prepare a 1 mg/mL polymer solution. These aniomer and catiomer solution were subjected to vortex-

mixing at a molar ratio of carboxyl groups to amine groups in the side chains with a range of 0.85 ≦ [carboxyl]/[amine] (C/A) ≦ 1.15. The mixed solution was vortexed (2,000 rpm, 2 minutes) to formPIC/m. The solution of EDC/HCl (10 molar equivalent to carboxyl units of PEG-PAsp) with a concentration of 10 mg/mL in 10 mM PB (pH 7.4, 0 mM NaCl) was added and then reacted at room temperature overnight for cross-linking the PIC core. The excess EDC/HCl and block copolymers were removed by the purification *via* ultrafiltration with VIVA SPIN 6 (Satrious stedium Biotech GmbH, Goettingen, Germany) [MWCO: 100,000 Da]. During the purification process, the solvent was replaced with deionized water (for SLS measurements), 10 mM PB (pH 7.4, 0 mM NaCl, for DLS and ELS measurements), 10 mM deuterium PB (pD 7.0, 0 mM NaCl, for SAXS measurements) or D-PBS (-) (for animal experiments).

Characterization of PIC/m

Dynamic light scattering (DLS) measurement

The size and corresponding of PIC/m were evaluated by conducting DLS measurement at room temperature in 10 mM PB (pH 7.4, 0 mM NaCl) using a Zatasizer Nano ZS90 (Marvern Instruments Ltd., Worcestershire, UK) equipped with a diode-pumped laser (532 nm). The intensity-averaged hydrodynamic diameter and polydispersity index (PdI) were derived according to the cumulant method. The surface zeta potential of PIC/m was evaluated by conducting electrophoretic light scattering (ELS) measurement using a Zatasizer Nano ZS90 at room temperature. PIC/m samples were prepared n 10 mM PB (pH 7.4, 0 mM NaCl) and 10 mM acetic acid buffer (pH 4.0, 0 mM NaCl), and then cross-linked and purified by ultrafiltration.

Static light scattering (SLS) measurement

The static light scattering (SLS) measurement was done using a DLS-8000 instrument (Otsuka Electronics, Osaka, Japan). Vertically polarized light of 633 nm wavelength from a He-Ne laser was used as the incident beam. The increments of refractive index, dn/dc, of the solutions were measured using a DRM-3000 double beam differential refractometer (Otsuka Electronics, Osaka, Japan). All measurements were carried out at room temperature. Molecular weight of each PIC/m was calculated from obtained Zimm plots as previously described [2].

^{1}H-NMR measurement of PIC/m

PIC/m were prepared with a concentration of 10 mg/mL in 10 mM deuterated phosphate buffer (PB, pD 7.0), and then ^{1}H-NMR measurement was conducted using JEOL ECS400 (JEOL Ltd., Tokyo, Japan) at room temperature. It has been reported that the peak intensity of poly(amino acid) protons appears lower than expected from the structure of building block copolymers due to the restrained mobility of atoms by assembled into the core of micelles [3-5]. Detected proton

ratio of PIC/m were calculated by comparing the peak area ratio to free block copolymers (PEG-PAsp and PEG-P(Asp-AP)) using the methylene protons of PEG as a reference.

4. ***In vivo*** **experiment**

Animals

BALB/c mice (female; 5-weeks-old) were purchased from Charles River Laboratories Japan, Inc. (Yokohama, Japan). All animal experiments were carried out in accordance with the guidelines at The University of Tokyo and Innovation Center of NanoMedicine.

Biodistribution study

Balb/c mice (n = 3, female, 7 weeks old) were intravenously injected with 200 μL of 1 mg/mL cy5-labeled PIC micelle in D-PBS(-). And then, the mice were sacrificed 60 min after sample administration and the excess blood was washed by perfusion with D-PBS(-). Blood was collected from the interior vena cava, heparinized and centrifuged to obtain plasma. The liver, spleen, kidney, lung, heart and brain were excised, washed with D-PBS(-), weighed after removing excess fluid and homogenized with cell lysis buffer using Multi Beads Shocker MBX (Yasui Kikai, Osaka, Japan). The accumulated amount of PIC/m was quantified by fluorescence measurement using an Infinite M2000 Pro spectrophotometer (Tecan, Mannedorf, Switzerland).

Intravital real-time confocal laser-scanning microscopy

All the intravital observation were performed using A1R confocal laser scanning microscope (Nikon Co., Tokyo, Japan) connected to an upright Eclipse FN1 (Nikon Co.). The 200 μL of cy5-labeled PIC micelle in D-PBS(-) was intravenously administrated to mice (Balb/c, female, 7 weeks old) under the anesthetized with 2.5 % isoflurane using a NARCOBIT-E Univentor 400 Anesthesia Unit (Natsume Seisakusho Co. Ltd., Tokyo, Japan), and then real-time observation was conducted continuously. The anesthetized mice were placed onto Thermoplate (Tokai Hit Co. Ltd., Shizuoka, Japan) with the temperature set 37 ℃. Cy5 was excited with a 640-nm laser and detected using 700-50-nm bandpass emission filter. A 20× objective lens was used for earlobe imaging and 40× objective lens was used for liver imaging. Obtained mages were processed using NIS-Elements software (Nikon Co.).

Intravital observation of blood vessel and liver

The blood circulation profile of cy5-labeled PIC micelles was evaluated by measuring the fluorescent intensity of the blood vessel lumen in the earlobe for 60 minutes continuously after the intravenous administration of samples to the mice as previously described [6]. The fluorescent intensity in the region of interest (ROI) in the vein was measured, followed by the subtraction of

the background fluorescent intensity in ROI before the sample administration. The intensity value was standardized with the maximum fluorescent intensity in ROI during the observation.

For the liver imaging, 10 μg of eFluor 450-comjugated PECAM-1 (CD31) was intravenously injected to the mice 30 minutes before the PIC micelle administration in order to visualize sinusoidal walls by exciting eFuor 450 using a 405 nm laser and detecting 450/50-nm emission filter. Liver imaging was conducted for 60 minutes after the intravenous sample administration as previously reported [7].

Supplementary References

1. Koide, A.; Kishimura, A.; Osada, K.; Jang, W.-D.; Yamasaki, Y.; Kataoka, K. Semipermeable Polymer Vesicle (PICsome) Self-Assembled in Aqueous Medium from a Pair of Oppositely Charged Block Copolymers: Physiologically Stable Micro-/Nanocontainers of Water-Soluble Macromolecules. *J Am Chem Soc* **2006**, *128*, 5988-5989, doi:10.1021/ja057993r.
2. Harada, A.; Kataoka, K. Effect of Charged Segment Length on Physicochemical Properties of Core−Shell Type Polyion Complex Micelles from Block Ionomers. *Macromolecules* **2003**, *36*, 4995-5001, doi:10.1021/ma025737i.
3. Chandran, T.; Katragadda, U.; Teng, Q.; Tan, C. Design and evaluation of micellar nanocarriers for 17-allyamino-17-demethoxygeldanamycin (17-AAG). *Int J Pharm* **2010**, *392*, 170-177, doi: 10.1016/j.ijpharm.2010.03.056.
4. Endres, T.K.; Beck-Broichsitter, M.; Samsonova, O.; Renette, T.; Kissel, T.H. Self-assembled biodegradable amphiphilic PEG–PCL–lPEI triblock copolymers at the borderline between micelles and nanoparticles designed for drug and gene delivery. *Biomaterials* **2011**, *32*, 7721-7731, doi:10.1016/j.biomaterials.2011.06.064.
5. Tao, A.; Huang, G.L.; Igarashi, K.; Hong, T.; Liao, S.; Stellacci, F.; Matsumoto, Y.; Yamasoba, T.; Kataoka, K.; Cabral, H. Polymeric Micelles Loading Proteins through Concurrent Ion Complexation and pH-Cleavable Covalent Bonding for In Vivo Delivery. *Macromol Biosci* **2020**, *20*, e1900161, doi:10.1002/mabi.201900161.
6. Watanabe, S.; Hayashi, K.; Toh, K.; Kim, H.J.; Liu, X.; Chaya, H.; Fukushima, S.; Katsushima, K.; Kondo, Y.; Uchida, S., et al. In vivo rendezvous of small nucleic acid drugs with charge-matched block catiomers to target cancers. *Nat Commun* **2019**, *10*, 1894, doi:10.1038/s41467-019-09856-w.
7. Dirisala, A.; Uchida, S.; Toh, K.; Li, J.; Osawa, S.; Tockary, T.A.; Liu, X.; Abbasi, S.; Hayashi, K.; Mochida, Y., et al. Transient stealth coating of liver sinusoidal wall by anchoring two-armed PEG for retargeting nanomedicines. *Sci Adv* **2020**, *6*, eabb8133,

doi:10.1126/sciadv.abb8133.
